# Cryptococcal meningoencephalitis in a patient with hyper immunoglobulin M (IgM) syndrome: a case report

**DOI:** 10.1186/1756-0500-7-566

**Published:** 2014-08-26

**Authors:** Luís Malheiro, Daniela Lazzara, Sandra Xerinda, Maria Dolores Pinheiro, António Sarmento

**Affiliations:** Infectious Disease Department–Nephrology Research Development Unit (FCT-725), Faculty of Medicine, University of Porto, Centro Hospitalar São João, Porto, Portugal; Serviço de Patologia Clínica, Centro Hospitalar São João, Porto, Portugal

**Keywords:** Hyper immunoglobulin M syndrome, *Cryptococcus neoformans*, Meningitis, Meningoencephalitis, Diplopia

## Abstract

**Background:**

Cryptococcal meningoencephalitis is an opportunistic infection that predominantly affects immunocompromised patients. Hyper immunoglobulin M syndrome is a primary immunodeficiency syndrome that increases susceptibility to several opportunistic infections. Here, we report a case of cryptococcal meningoencephalitis in the context of hyper immunoglobulin M syndrome, a situation that has been reported very few times and whose management is not clearly defined. We describe our management of this case and the outcome of the patient to help in future similar situations.

**Case presentation:**

The patient is a 19-year-old Caucasian male student diagnosed with X-linked hyper immunoglobulin M syndrome and treated chronically with weekly intravenous immunoglobulin and daily sulfamethoxazole-trimethoprim. He was admitted to the infectious diseases ward because of headache, diplopia and a cerebral-spinal fluid analysis revealing cryptococcal meningoencephalitis. The patient was treated with liposomal amphotericin and flucytosine with a favorable outcome. Maintenance therapy with fluconazole has continued and will be sustained for 6 months following his upcoming bone marrow transplantation.

**Conclusion:**

Monitoring for cryptococcal meningoencephalitis should be considered in patients with primary immunodeficiencies, as clinical manifestations may go unnoticed. In these patients, it is expected that chronic treatment with fluconazole will be the only treatment that will prevent reinfection or reactivation, and therefore should be kept at least until bone marrow transplant, the only curative treatment, is performed. It may, however, lead to intolerable side effects and hepatic toxicity.

## Background

**Figure 1:**
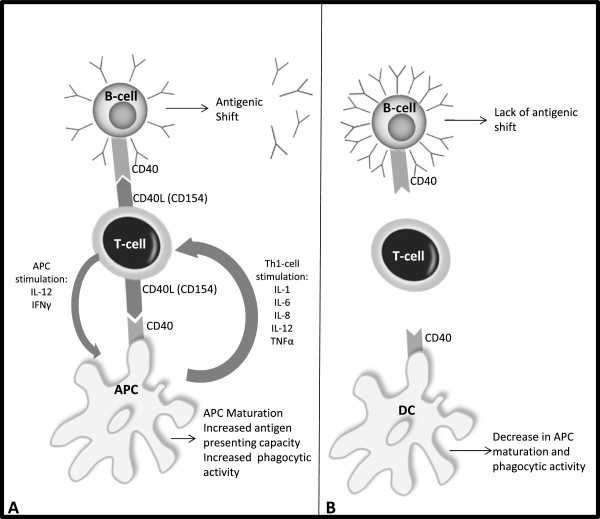
**Diagram of the immunologic effects of hyper IgM syndrome. A)** In a healthy individual, the CD154 (CD40L)-CD40 interaction is essential for antigen-presenting cell (APC) maturation and B-cell antigenic shift towards the production of IgG, IgA and IgE. APC maturation leads to increased antigen-presenting capacity and phagocytic activity, as well as interleukin (IL) production that further stimulates T helper-1 (Th1) cell differentiation. **B)** In X-linked hyper IgM syndrome, CD40L mutations decrease the capacity of T-cells to differentiate and interact with other immune system cells, increasing susceptibility to opportunistic infections.

Hyper immunoglobulin M (HIGM) syndrome is a primary immunodeficiency (PID) that was described for the first time in 1961 [1]. It is a well-known PID, classified as a B-cell immunodeficiency and characterized by normal to high immunoglobulin (Ig) M levels and absent IgG, IgA and IgE due to an impairment of class switching recombination [2]. It results from a mutation of the CD154 gene that encodes for a T-cell membrane protein (CD40 ligand), which is normally able to activate CD40 in B-cells and antigen-presenting cells (APC). Impairment of this interaction leads to a lack of immunoglobulin class switching in B-cells, and a reduced ability of monocytes to induce allogeneic T-cell proliferation (Figure 1). The net result is an increased susceptibility to opportunistic infections by *Pneumocystis jirovecii*, *Cryptosporidium spp.*, and other intracellular organisms, and a higher rate of bacterial and viral disseminated infections. In the majority of cases, the condition has an X-linked inheritance, as is the case with this report of a young adult male with *Cryptococcus neoformans* meningoencephalitis.

## Case presentation

On April 29th 2013, a 19-year-old Caucasian male student was observed in the emergency department of our hospital complaining of diplopia, which started 2 weeks prior, and a mild frontal, bilateral, dull headache, persisting over the previous 2 days. He denied recently experiencing any fever, vomiting, nausea or any focal neurological signs. On physical examination, only a bilateral mild papillary border elevation was perceived by fundoscopy. Blood tests revealed the following measurements: hemoglobin, 14.8 g/dL; leucocytes, 9.02 × 10^9^/L (neutrophils, 60%; lymphocytes, 21%); and platelets, 129 × 10^9^/L. Blood chemistry showed normal renal and hepatic function, but with a C-reactive protein level of 17.3 mg/L (normal: <10 mg/L). The computed tomography (CT) scan of the patient’s brain was normal. A lumbar puncture (LP) was performed, which resulted in the release of clear cerebral-spinal fluid (CSF) and had an opening pressure of 33 cm H_2_O (normal: 7–14 cm H_2_O). Analysis of the CSF showed 120 leucocytes per cubic millimeter with 94.2% mononuclear cells, glucose levels <50% (43 mg/dL) of seric value (normal: >50% of seric value), and 0.87 mg/mL of proteins (normal: <0.5 mg/mL). The CSF cryptococcal antigen test was negative. The patient was treated empirically with ampicillin and ceftriaxone.

The patient’s medical history indicated that he had been diagnosed with X-linked HIGM syndrome at the age of 6 months in the setting of a severe *Pneumocystis jirovecii* pneumonia with secondary bronchopulmonary dysplasia. He has been treated chronically with weekly intravenous immunoglobulin (IVIg) and daily sulfamethoxazole-trimethoprim since 6 months of age. In spite of these treatments, he had several lower respiratory tract infections during childhood. His parents are asymptomatic, but his mother is a CD154 mutation carrier.

**Figure 2:**
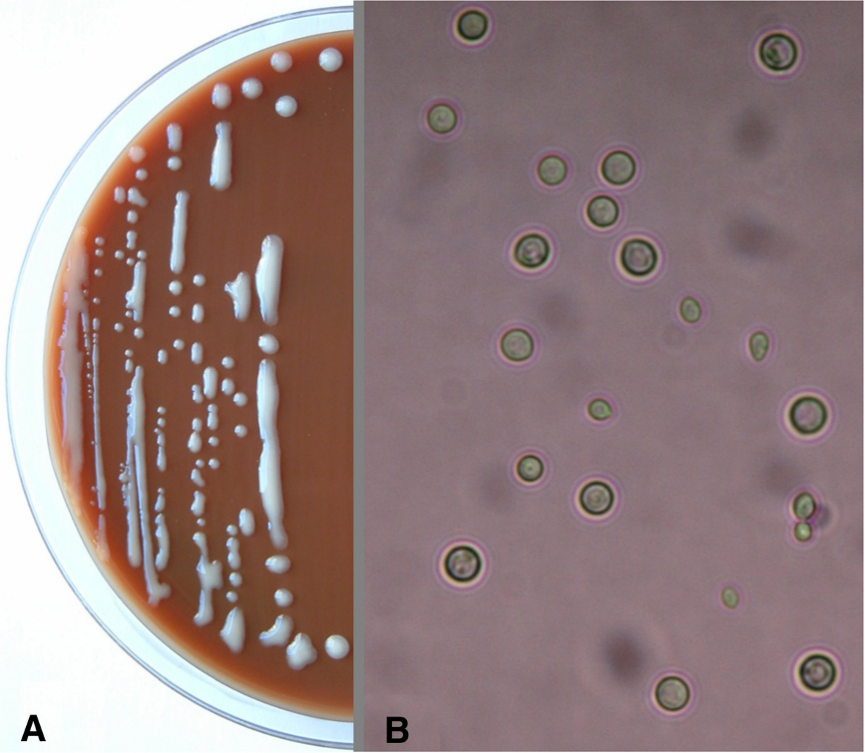
**Microscopic photographs of** ***Cryptococcus neoformans*** **. A)** Gray, mucoid colonies, highly suspicious of Cryptococcus spp., growing in solid media. **B)** Direct examination of colonies in suspension.

The CSF sample obtained on admission was sent to the microbiology laboratory, where a Gram-stained smear, a direct India ink exam, and blood and chocolate agar cultures at 37°C and 5% CO_2_ were performed. While the results from the Gram stain and direct exam were negative, after 24 hours of incubation, gray, mucoid colonies, highly suspicious of *Cryptococcus spp.*, were growing (Figure 2A). A direct examination of the colonies suspended in distilled water revealed round cells in a range of sizes, suggestive of yeast (Figure 2B). The identification of *Cryptococcus neoformans* was provided by a Vitek2 System™ (bioMerieux™) and confirmed by matrix-assisted laser desorption-ionization time-of-flight (Maldi-Tof™) mass spectrometry with Vitek MS™ (bioMerieux™). The serum cryptococcal antigen titer was 1:64. Polymerase chain reaction (PCR) assays testing for *Mycobacterium tuberculosis* and *Listeria monocytogenes* were negative.

Following these findings, the empirical treatment with ampicillin and ceftriaxone was suspended and treatment with liposomal amphotericin and flucytosine was begun.

Two days later, a second CSF sample, with 104 leucocytes per cubic millimeter (57% lymphocytes and 7% neutrophils) and an opening pressure of 22 cm H_2_O was processed as described above. In this sample, the result from the India ink exam was positive and the yeast grown on the agar media were identified as *Cryptococcus neoformans* by Vitek MS™. The CSF cryptococcal antigen test was not repeated. The blood that had been collected upon admission was microbiologically negative.

The patient’s symptoms cleared after 7 days of treatment. Because of symptomatic improvement and the absence of increased intracranial pressure, regular evacuative LPs were not performed. After 2 weeks of treatment, a LP was repeated, yielding negative results from an India ink stain, Gram stain and culture. The cryptococcal serum antigen titer was 1:32. Treatment was changed to fluconazole (400 mg/day). During the length of his 18-day admittance, the patient maintained his usual prophylactic sulfamethoxazole-trimethoprim treatment and at discharge he was asymptomatic, with a normal physical exam. The patient remained on maintenance treatment for *C. neoformans*.

## Discussion

*Cryptococcus neoformans* is an encapsulated yeast found worldwide that is isolated predominantly from pigeon droppings and soil contaminated with avian excreta [3]. It is not unusual for humans to come into contact with it early in life, and a majority of children are likely to have been exposed by the age of 5. This situation probably applies to our patient, as he was raised in a rural area.

Inhalation of the yeast is the most frequent way of acquiring this infection. From the lungs the fungal cells may spread hematogenously to the brain, causing life-threatening meningitis or meningoencephalitis, or even to other organs. The disease can vary from localized to disseminated and from acute to chronic depending on the state of the patient’s immune system, which may be impaired by malignancy, immunosuppressive therapies, or acquired/primary immunosuppression syndromes, as is the case for the patient described in this report.

There have been several attempts to gather information about X-linked HIGM syndrome, though its rarity makes the development of a comprehensive clinical picture of this disease hard to achieve.

X-linked HIGM syndrome is expected to increase susceptibility to *C. neoformans*, as it has been shown to do *in vitro* [4], although the *in vivo* incidence is not as great as expected, evidenced by the fact that only a few cases have been reported [5–8]. IVIg therapy, used widely in these patients from a young age, significantly decreases the frequency of lower respiratory tract infections and severe infections; however, it does not change the frequency of non-respiratory or upper respiratory infections [9].

In this patient the symptoms were less intense than expected. Clinically, there were no major central nervous system symptoms, apart from diplopia for far-sighted focus, which lasted for 2 weeks, and headache for the 2 days before admittance. There were no findings in his blood that suggested an infection, and if it were not for his medical history and high clinical suspicion, a LP would not have been performed. Nonetheless, a cerebral CT scan should always be done in a young patient presenting with headache and neurologic focal signs for a long period. This lack of symptoms may reveal a decreased immune response to *Cryptococcus spp*., as it was shown that *in vitro* the CD154-CD40 interaction is essential for the secretion of IL-12 and IFNγ in response to this organism [10]. If these cytokines are not produced, the macrophages cannot activate T-cells through antigen-presenting dendritic cells, decreasing the rates of Th1 generation normally responsible for stimulating phagocytic activity towards fungal infections (Figure 1). As a consequence, the immune reaction will be less severe, with reduced immune activation and migration and fewer symptoms typically due to meningeal inflammation.

Amphotericin B and Flucytosine, suggested by current practice guidelines, were effective therapies against *Cryptococcus neoformans* as shown by the clinical improvement and the negative lumbar puncture after 2 weeks of combined treatment [11]. Although this treatment was effective, there is no consensus on the appropriate duration for maintenance therapy. In the HIV setting, there is evidence that maintenance therapy should be kept until an immunologic improvement, characterized by CD4^+^ T-cells > 100/μL for more than 3 months, is achieved [12]. However, in the X-linked HIGM setting, there is no evidence indicating whether to continue lifelong maintenance therapy or to stop after a short treatment. Patients with this disease are young, and lifelong therapy with fluconazole may lead to several side effects that can become intolerable and lead to hepatic toxicity. There are no methods, apart from bone marrow transplant (BMT), that can provide immune recovery for these patients; therefore, if they develop cryptococcal meningoencephalitis under treatment with IVIg, we expect that fluconazole will be the only treatment that will prevent reinfection or reactivation.

Recently, evidence that allogeneic BMT may lead to correction of the immunodeficiency, with varying degrees of success, has given new perspectives on this condition [13]. Our patient has been recommended to undergo BMT and continue maintenance therapy with fluconazole until he is healthy enough to undergo the procedure. However, after the procedure he will be exposed to a new serious immunodepression because of the necessary post-transplant treatment with immunosuppressors, and a relapse of the disease is possible. There are recommendations for patients that develop cryptococcal meningoencephalitis during BMT, but none for patients with a previous history of the disease, so the choice to keep maintenance therapy for 6–12 months after the procedure is debatable.

## Conclusion

X-linked HIGM syndrome is characterized by a high incidence of opportunistic infections with an unfavorable outcome, despite regular substitution therapy with IVIg. However, outcomes will likely be improved with additional therapies, like trimethoprim/sulfamethoxazole prophylaxis, and close follow-up. If these methods are successful, an older population of X-linked HIGM patients will develop in time, and new opportunistic infections that we are unaware of could also appear. Infections with *Cryptococcus spp.* may be more frequent than described and deserve further studies to determine treatment outcomes and duration.

## Consent

Written informed consent was obtained from the patient for publication of this case report and any accompanying images. A copy of the written consent is available for review by the Editor-in-Chief of this journal.
